# Mechanisms of Moral Injury Following Military Sexual Trauma and Combat in Post-9/11 U.S. War Veterans

**DOI:** 10.3389/fpsyt.2018.00520

**Published:** 2018-11-02

**Authors:** Sheila B. Frankfurt, Bryann B. DeBeer, Sandra B. Morissette, Nathan A. Kimbrel, Heidi La Bash, Eric C. Meyer

**Affiliations:** ^1^VISN 17 Center of Excellence for Research on Returning War Veterans, United States Department of Veterans Affairs, Waco, TX, United States; ^2^Central Texas Veterans Health Care System, Temple, TX, United States; ^3^College of Medicine, Texas A&M University Health Science Center, College Station, TX, United States; ^4^The University of Texas at San Antonio, San Antonio, TX, United States; ^5^Durham VA Medical Center, Durham, NC, United States; ^6^Mental Illness Research, Education and Clinical Centers MIRECC (VA), Durham, NC, United States; ^7^Department of Psychiatry and Behavioral Sciences, Duke University Medical Center, Durham, NC, United States; ^8^National Center for PTSD, VA Palo Alto Healthcare System, Palo Alto, CA, United States; ^9^Department of Psychology and Neuroscience, Baylor University, Waco, TX, United States

**Keywords:** moral injury, military sexual trauma, veteran, posttraumatic stress disorder, depression, guilt, shame, combat trauma

## Abstract

**Objective:** Moral injury may result from *perpetration-based* and *betrayal-based acts* that violate deeply held norms; however, researchers and clinicians have little guidance about the moral injury syndrome's specific developmental pathways following morally injurious events. The present study's objective was to examine the direct and indirect pathways proposed in a frequently cited model of moral injury (1) in relation to two types of military-related traumas [experiencing military sexual trauma (MST) and combat exposure].

**Methods:** Secondary analyses were conducted within a sample of post-9/11 veterans at a Southwestern Veterans Health Care System (*N* = 310) across two time-points. Structural equation modeling tested the direct and indirect pathways from MST and combat to a PTSD-depression factor via betrayal, perpetration, guilt, and shame.

**Results:** Betrayal accounted for the association between MST and PTSD-depression (β = 0.10, *p* < 0.01, 95% CI = 0.01 − 0.11) and perpetration accounted for the association between combat and PTSD-depression (β = 0.07, *p* < 0.05, 95% CI = 0.02 − 0.14). The indirect path from combat to shame to PTSD-depression was significant (β = 0.16, *p* < 0.01, 95% CI = 0.07 − 0.28) but the path through guilt was not. The specific indirect paths through perpetration or betrayal to shame or guilt were non-significant.

**Conclusions:** Betrayal and perpetration are associated with PTSD-depression following MST and combat. Results suggest multiple pathways of moral injury development following different military traumas and morally injurious events. Implications for moral injury conceptualization and treatment are discussed.

## Introduction

Moral injury describes the unique psychological harm of acting, failing to prevent, or witnessing actions that transgress one's deeply held values (i.e., perpetration-based morally injurious events) or being betrayed by a trusted authority figure in a high stakes situation [betrayal-based morally injurious events (1, 2)]. It was originally conceptualized to help account for the poorer functioning among combat veterans whose worst traumas involved acts of commission (e.g., killing) or omission (e.g., failing to prevent atrocities) over those whose worst traumas were life threat-based (3). Subsequently, Stein et al. (4) proposed that the construct be expanded to include noncombat betrayal-based experiences such as experiencing military sexual trauma (MST). Moral injury is thought to develop when veterans are unable to integrate the memories of their morally injurious events with their self-schemas and thus experience unresolved inner conflicts or moral dilemmas. These inner conflicts corrode their sense of self and engender guilt, shame, and rage (i.e., mechanisms of moral injury), which lead to the moral injury syndrome: depression, re-experiencing and avoidance trauma symptoms, substance abuse, spiritual/religious decline, and suicide (1, 3, 5).

There are a number of open questions in the moral injury field that directly impact clinicians' and researchers' ability to develop evidence-based, conceptually grounded assessment tools and psychotherapies. The boundary conditions of the perpetration-based or betrayal-based morally injurious event categories, (i.e., what counts as exposure to a potentially morally injurious event) are ill-defined and debated. There is little specific formulation about the relationships among perpetration and betrayal and the mechanisms of moral injury development such as guilt and shame, or their association with moral injury outcomes such as depression and PTSD. Thus, there is a great deal of unexamined heterogeneity in clinical presentations of moral injury and few empirical data to guide clinicians and researchers in developing either idiographic case conceptualizations of particular veterans or nomothetic evidence-based interventions for moral injury.

The current study tested whether the pathways that lead from two military traumas—combat exposure and MST—to PTSD and depression conformed to the moral injury framework. Specifically, we tested whether appraising MST and combat as either perpetration-based or betrayal-based morally injurious events leads to PTSD and depression, and whether guilt and shame accounted for any association between the military traumas, morally injurious events, and PTSD and depression. We conceptualized the betrayal-based and perpetration-based morally injurious events and guilt and shame as multiple mediating layers of the moral injury syndrome (here modeled as PTSD and depression) (1). Our goal was to identify potential modifiable mechanisms of moral injury that can be used to develop clinical profiles and, eventually, targeted treatments to help veterans relieve the burden of their moral injuries.

### Military sexual trauma and moral injury

MST refers to any experience of sexual assault or repeated, threatening sexual harassment that occurred during a veteran's military service. MST can occur when the veteran was on or off duty, as well as on or off base, and could have been perpetrated by another service member or a civilian [definition from Federal law; Title 38 U.S. Code 1720D (6)]. A recent MST prevalence meta-analysis estimated that 24% of women and 2% of men reported military sexual assault and 53% of women and 9% of men reported military sexual harassment (7). MST is a risk factor for PTSD and depression, two psychological outcomes included in the moral injury syndrome, in both male and female post-9/11 veterans (8, 9).

#### Betrayal

Some moral injury researchers suggest that experiencing MST can be a morally injurious event because it may involve significant perceived betrayal by fellow service members (via within-rank violence) and military leadership in some circumstances (4, 10–12). In a sample of Army National Guard Soldiers redeploying from Afghanistan (*N* = 935), lifetime history of unwanted sexual activity (including pre-military and military time periods) was significantly correlated with perceived betrayal but not perceived perpetration (10). Two qualitative studies of morally injurious events in combat veterans' trauma narratives (4, 11) conceptualized MST as manifestations of within-rank violence or moral injury by others; however, neither coding guidelines nor examples of MST-related trauma narratives were provided. Thus, betrayal qualities of MST were difficult to evaluate. No studies have directly examined whether experiencing MST is associated with betrayal or if MST is associated with moral injury outcomes through its association with betrayal.

#### Shame and guilt

A rich clinical and empirical literature has addressed the shame- and guilt-based reactions to sexual trauma, albeit in the context of PTSD and not moral injury (13–16). Shame and guilt have been described as an inherent reaction to the social subordination and degradation of being sexually assaulted (13) or as reflecting the internalization of underlying negative beliefs about oneself as a result of the trauma (15). The extent to which veterans feel shame vs. guilt after MST or betrayals, and whether shame vs. guilt accounts for any association between betrayal and PTSD and depression after MST is an open question.

### Combat and moral injury

Combat is the primary context in which perpetration- and betrayal-based morally injurious events occur (3). Prototypical perpetration-based combat-related morally injurious events are killing enemies or non-combatants and participating in or failing to prevent excessive violence or atrocities. Among post-9/11 U.S. veterans, 40–65% of Army and Marine Iraq War veterans reported killing an enemy and ~15% of Army and ~30% of Marine Iraq War veterans reported killing a noncombatant [see (3) for a review]. The prevalence of morally injurious events have not been systematically assessed in post-9/11 veterans.

#### Perpetration and betrayal

Perpetration- and betrayal-based morally injurious events are commonly assessed using the Moral Injury Events Scale [MIES (17)], which asks about perpetration of morally troubling acts by oneself or others and being betrayed by others. In a nationally representative sample of U.S. combat veterans, 10% reported perpetrations committed by the self, 25% reported perpetrations committed by others, and 25% reported being betrayed (18). MIES scores predicted higher odds of a current mental disorders (generalized anxiety, PTSD, and depression) and current suicidal ideation. Overall, morally injurious events are correlated with both PTSD and depression in samples of combat-deployed Marines (17), combat-deployed Army National Guard soldiers (10), and mental health treatment-seeking active duty Airmen (10).

#### Shame and guilt

In the moral injury framework, the guilt and shame engendered by perpetrating morally injurious events are conceptualized as being, at some level, an appropriate and not irrational response (19, 20). Both guilt and shame may be salutary in that they may signal an intact conscience and promote prosocial reparative behavior and interpersonal reconnection (21, 22). A handful of studies have tested the associations between combat, guilt, and moral injury outcomes (23–26). Three found that combat-related guilt accounted for the association between perpetration-based combat experiences (e.g., killing in combat, participating in or observing atrocities) and moral injury-related outcomes such as PTSD, depression, or suicide (23, 24, 26). One found that perpetration was associated with state-based guilt, although guilt was not directly associated with PTSD (25). In general, few studies have distinguished between guilt and shame or attempted to parcel their relative contribution to outcomes following morally injurious events. Further, few studies have examined the associations among these variables over time.

### Summary and current study

The current study's aim was to test proposed developmental mechanisms (guilt, shame) of moral injury (PTSD, depression) following two types of military trauma exposure (MST, combat) and two types of morally injurious events (betrayal, perpetration) within the context of a longitudinal parent study. Our goal was to provide empirically grounded preliminary guidance regarding clinical profiles of moral injury and potential modifiable factors that could be targeted in moral injury treatment. Overall, there are few exhaustive tests of the multiple intervening mechanisms in the moral injury model (e.g., testing relations from military trauma to appraisals of perpetration or betrayal to guilt and shame to moral injury outcomes). Few empirical data are available on experiencing MST within a moral injury framework and no studies have assessed whether experiencing MST is associated with PTSD and depression via betrayal. Likewise, few attempts have been made to integrate conceptualizations of perpetration- and betrayal-related guilt and shame within existing models of posttraumatic shame and guilt. Because exposure to combat trauma and MST is not mutually exclusive and both experiences may contribute to veterans' cumulative PTSD and depression (27–29), we included both types of military traumas in the current study to enhance ecological validity.

**Figure 1:**
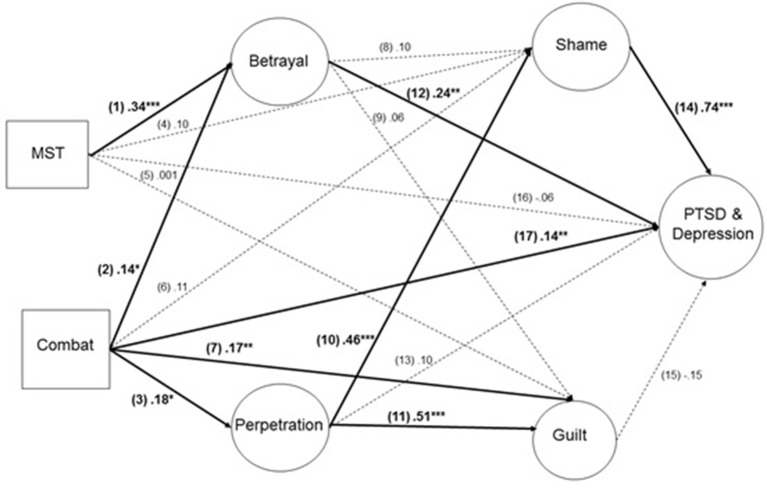
Standardized path coefficients reported. Solid lines indicate significant paths and dashed lines indicate non-significant paths (p > 0.05). ^*^p < 0.05, ^**^p < 0.001, ^***^p < 0.0001 (2-tailed).

The key pathways representing the moral injury model were the indirect paths: (I) from MST through betrayal to a composite latent factor reflecting PTSD-depression, (II) from combat through betrayal to PTSD-depression, and (III) from combat through perpetration to PTSD-depression. The predicted direct pathways were numerically labeled to facilitate interpretation, as follows (see Figure 1). We predicted direct pathways from MST to betrayal (1), from combat to betrayal (2), and from combat to perpetration (3). We did not model the pathways from MST to perpetration because our measure of MST involved victimization only and thus a pathway through perpetration was neither conceptually nor clinically appropriate. To account for the guilt- and shame-based PTSD framework, we modeled the direct paths from MST to shame (4), from MST to guilt (5), from combat to shame (6), and from combat to guilt (7). We modeled the paths from betrayal to shame (8) and guilt (9), and from perpetration to shame (10) and guilt (11); these were considered exploratory. Because affective reactions to perpetration and betrayal other than shame and guilt may contribute to PTSD-depression [e.g., anger (25)], we also modeled the direct paths from betrayal to PTSD-depression (12) and perpetration to PTSD-depression (13). Given the established relations from guilt and shame to both depression (30) and PTSD, we modeled the paths from shame to PTSD-depression (14) and guilt to PTSD-depression (15). Based on the accumulated body of trauma literature, we expected significant direct pathways from MST to PTSD-depression (16) and from combat exposure to PTSD-depression (17).

## Materials and methods

### Participants and procedures

This study was carried out in accordance with the recommendations of Central Texas Veterans Healthcare System Institutional Review Board. All subjects gave written informed consent in accordance with the Declaration of Helsinki consent prior to beginning the face-to-face baseline assessment. U.S. post-9/11 war veterans participated in a parent study of potentially modifiable psychosocial factors impacting adjustment over time following warzone service. Veterans were recruited using flyers posted throughout the medical center, letters mailed to a randomly selected listed of post-9/11 veterans enrolled in the local VA healthcare system, and through health provider referrals. Although veterans must have been enrolled in the VA healthcare system to participate, actual treatment seeking was variable and was not a requirement of eligibility. Specific populations (women, veterans with PTSD and depression) were over-sampled through targeted mailings; diagnoses for oversampling were based on the electronic medical record. Veterans were excluded if they had plans to relocate within the subsequent 4 months or met criteria for a psychotic or bipolar disorder. If veterans were receiving psychiatric care at the time of the baseline assessment, they were required to have reached treatment stabilization criteria for at least 3 months. Veterans were included in the current study if they were administered both the MST measure [DRRI; (31)] and the Moral Injury Events Scale [MIES (17)] (*N* = 310). Data were gathered at two time points: MST and combat exposure were assessed at baseline; betrayal, transgression, shame, guilt, and PTSD, and depression were assessed 16 months later. Retention between time one and time two was extremely high (87%). Participants completed the self-report questionnaires at a VA medical center at baseline and by mail or online at the 16-month follow-up.

### Measures

The Mini International Neuropsychiatric Interview (32) was used at baseline to screen for the excluded diagnoses of psychotic or bipolar disorders (Diagnostic and Statistical Manual for Mental Health Disorders, 4th Edition; *DSM-IV*; APA, 2000).

*Combat Exposure* was measured using 18-item Full Combat Exposure Scale (33). Items are rated on a 5-point scale ranging from 0 (*never*) to 4 (*10*+ *times*); the total summed score was used. In the current study, internal consistency using Cronbach's α was 0.92.

*MST* was measured using the 8-item Sexual Harassment Scale on the Deployment Risk and Resilience Inventory-2 (31). Items were scored on a 4-point Likert-style scale (1 = *Never* to 4 = *Many times*). In the validation sample of Iraq/Afghanistan veterans, internal consistency was α = 0.86 (31). In the current sample, internal consistency was α = 0.89. MST summed scores were used.

*Betrayal* and *perpetration* were measured using the 9-item Moral Injury Events Scale (MIES; 16). Items are rated on a 6-point Likert-style scale (1 = *Strongly agree* to 6 = *Strongly disagree*) scale. In the validation sample of combat-deployed Marines, internal consistency for the full measure was Cronbach's α = 0.90 (17). In the current sample, the internal consistency of the betrayal factor was Cronbach's α = 0.85; and of the perpetration factor was Cronbach's α = 0.94.

*Shame* and *guilt* were measured using the 10-item State Shame and Guilt Scale (34). Respondents rated five guilt-related items (e.g., *I feel remorse, regret*) and five shame-related items (e.g., *I feel that I am a bad person*) on a 5-point Likert style scale (1 = *Not feeling this way at all* to 5 = *Feeling this way very strongly*) scale (see Table **2** for item factor loadings). Estimates of internal consistency in a prior study of veterans were Cronbach's α = 0.69 for the guilt subscale and Cronbach's α = 0.76 for the shame subscale (35). In the current sample, guilt subscale Cronbach's α was 0.91 and shame subscale Cronbach's α was 0.91.

*PTSD symptoms* were measured at the 16-month follow-up assessment using the 20-item Posttraumatic Stress Disorder Checklist for DSM-5 [PCL-5 (36)]. Participants rated how much they were bothered by each of the 20 DSM-5 PTSD symptoms over the past month in relation to stressful military experiences on a 4-point Likert-style scale (0 = *Not at all* to 4 = *Extremely*). In the PCL-5 validation study, internal consistency was Cronbach's α = 0.95 with a clinical cut-off of 33 to indicate probable PTSD (37). In the current study, internal consistency was Cronbach's α = 0.97.

*Depressive symptoms* were measured at the 16-month follow-up assessment using the 9-item Patient Health Questionnaire-9 (38). Respondents indicated how often they have been bothered by symptoms of depression over the past 2 weeks on a 3-point Likert scale (0 = *not at all* to 3 = *nearly every day*). A PHQ-9 score of 10 is suggested as the cut-off for moderate depression (38). In the current study, internal consistency was Cronbach's α = 0.91.

### Data analysis plan

Preliminary analysis of study variables was conducted before testing our primary and secondary models. Approximately half of the items on the MST measure were skewed (0.99–5.06) and leptokurtic (-0.67–25.86) and so summed MST total scores were used. The summed total MST score was not significantly skewed (2.47) and was leptokurtic (6.05); however, non-normality is unlikely to affect parameter estimates when maximum likelihood estimation is used and sample sizes are larger than *N* = 100 (39).

The pattern of correlations among study variables was examined (Table 1). Next, we tested the full measurement model. Lastly, we tested the structural model assessing the direct and indirect pathways from MST and combat exposure to PTSD-depression via perpetration and betrayal, and guilt and shame. Model fit was determined using four indices: χ^2^ test of model fit, comparative fit index (CFI), Tucker-Lewis fit index (TLI), the root mean square error of approximation (RMSEA), and the standardized root mean square residual (SRMR). CFI and TLI values >0.90 and RMSEA and SRMR values of ≤0.08 are considered indices of good fit (40). Indirect paths were evaluated using the bias-corrected bootstrapped confidence intervals. SEM was conducted in Mplus Version 7.3, which estimated models using maximum likelihood estimation. All reported paths were standardized using the Mplus STDYX procedure.

**Table 1:** Descriptive statistics and correlations.

	**Mean**	**SD**	**1**.	**2**.	**3**.	**4**.	**5**.	**6**.	**7**.	**8**.
Military sexual trauma	10.44	4.51	–	−0.08	0.42^**^	0.26^**^	0.23^**^	0.13^*^	0.19^**^	0.22^**^
Combat	21.57	14.00	–	–	0.08	0.18^*^	0.18^**^	0.25^**^	0.21^**^	0.31^**^
Betrayal	10.24	4.93	–	–	–	0.59^**^	0.37^**^	0.34^**^	0.44^**^	0.49^**^
Perpetration	17.91	8.40	–	–	–	–	0.50^**^	0.51^**^	0.48^**^	0.56^**^
Shame	9.82	5.42	–	–	–	–	–	0.82^**^	0.75^**^	0.70^**^
Guilt	9.79	5.42	–	–	–	–	–	–	0.65^**^	0.64^**^
Depression	11.50	7.03	–	–	–	–	–	–	–	0.81^**^
PTSD	35.68	21.22	–	–	–	–	–	–	–	–

* p < 0.05;

** *p < 0.001 (2-tailed)*.

## Results

The current sample was mostly male (76%, *n* = 235) and middle-aged (*M* = 40.67, *SD* = 8.55). Racial diversity reflected the geographic area: 57% white (*n* = 177), 32% African American (*n* = 99), 5% Asian American (*n* = 15), 6% American Indian/Alaska native (*n* = 18), 1% Hawaiian/Pacific Islander (*n* = 3), and 3% other (*n* = 9). Hispanic veterans (19%, *n* = 60) were also well-represented. Participants tended to be married (66%, *n* = 205) with some college experience but no degree (45%, *n* = 138). On average, participants enlisted in military service at 20.93 years old (*SD* = 4.49) and the majority served in the Army (90.3%, *n* = 280) on active duty (96.8%, *n* = 300) for an average of 13.50 years (*SD* = 7.61). The modal number of deployments to Iraq or Afghanistan was 2 (*SD* = 1.11, range = 1–7); 86.8% of participants had deployed to Iraq (*n* = 269) and 30% (*n* = 93) had deployed to Afghanistan (categories not mutually exclusive). The average PCL-5 total score was 35.68 (*SD* = 21.22), and 54.4% of the sample had PCL-5 scores at or above the clinical cutoff for PTSD. The average PHQ-9 total score was 11.50 (*SD* = 7.03), which is in the moderate depression range.

In total, 42.3% of the sample (*n* = 131; 28.9% of men, *n* = 68; 83.8% of women, *n* = 62; 100% of transgender veterans, *n* = 1) reported at least one experience of MST. The most common were being subjected to crude and offensive sexual remarks (38.1%, *n* = 118) and having negative rumors spread about the veteran's sexual activities (22.3%, *n* = 70). For MST involving threat or coercion, 12.3% (*n* = 56) of veterans reported being pressured into sex involving use of a position of authority; 7.4% (*n* = 23) reported being offered a specific reward or special treatment for participation in sex; and 8.1% (*n* = 25) reported being threatened with retaliation for not being sexual cooperative. Significantly more women than men reported each type of MST. Nearly all participants (98.7%, *n* = 306) were exposed to some form of combat. The combat exposure measure assessed two prototypical perpetration-based acts: being directly responsible for the death of an enemy combatant (28.2%, *n* = 89) and being directly responsible for the death of a noncombatant (12%; *n* = 39).

### Measurement model

Latent variables in the primary measurement model were betrayal, perpetration, shame, guilt, and PTSD-depression. First, the full measurement model was tested. The unmodified measurement model fit poorly, $X_{\left(1070\right)}^{2}$ = 4959.30, *p* < 0.001, RMSEA = 0.09, CFI = 0.79, TLI = 0.78, SRMR = 0.07. Modification indices suggested correlating pairs of MIES items that were correlated in Nash et al. (17). PTSD and depression were modeled as one latent variable due to the high correlation between the PCL-5 and PHQ-9 and to reflect the moral injury syndrome. Modification indices suggested significant model fit improvement by correlating pairs of PTSD and depression symptom items that either assessed similar aspects of PTSD symptoms within the same PTSD diagnostic criterion (B-E) or were a pair of PCL-5 and PHQ-9 items that assessed difficulty falling asleep. Perpetration and betrayal were correlated, as were shame and guilt, due to shared measurement variance. The modified measurement model yielded adequate fit, $X_{\left(1060\right)}^{2}$ = 3510.73, *p* < 0.001, RMSEA = 0.07, CFI = 0.87, TLI = 0.86, SRMR = 0.06. See Table 2 for factor loadings and item and factor correlations.

**Table 2:** Factor loadings of measurement model.

**Factor**	**Measure**	**Variable**	**Loading**	**S.E**.
Betrayal	MIES	(7) I feel betrayed by leaders who I once trusted	0.75	0.04
		(8) I feel betrayed by fellow service members whom I once trusted	0.76	0.04
		(9) I feel betrayed by others outside the U.S military whom I once trusted	0.75	0.04
Perpetration	MIES	(1) I saw things that were morally wrong	0.49	0.05
		(2) I am troubled by having witnessed others' immoral acts	0.60	0.04
		(3) I acted in ways that violated my own moral code or values	0.80	0.03
		(4) I am troubled by having acted in ways that violated my morals	0.85	0.02
		(5) I violated my own morals by failing to do something…	0.87	0.02
		(6) I am troubled because I violated my morals by failing to do something…	0.88	0.02
Depression and PTSD	PHQ-9	(1) Little interest or pleasure in doing things	0.74	0.03
		(2) Feeling down, depressed, or hopeless	0.80	0.02
		(3) Trouble falling asleep, staying asleep or sleeping too much	0.63	0.03
		(4) Feeling tired or having little energy	0.67	0.03
		(5) Poor appetite or overeating	0.59	0.04
		(6) Feeling bad about yourself, feeling that you are a failure	0.75	0.03
		(7) Trouble concentrating	0.73	0.03
		(8) Moving or speaking noticeably slower or being so fidgety	0.66	0.03
		(9) Thinking that you would be better off dead	0.49	0.04
	PCL-5	(1) Repeated, disturbing, and unwanted memories	0.80	0.02
		(2) Repeated, disturbing dreams of the stressful experience	0.75	0.03
		(3) Suddenly feeling or acting as if it were actually happening again	0.76	0.02
		(4) Feeling very upset when something reminded you	0.83	0.02
		(5) Having strong physical reactions at reminders	0.80	0.02
		(6) Avoiding memories, thoughts, or feelings	0.76	0.03
		(7) Avoiding external reminders of the stressful experience	0.78	0.02
		(8) Trouble remembering important parts of the stressful experience	0.55	0.04
		(9) Having strong negative beliefs about yourself, people, world	0.77	0.02
		(10) Blaming yourself or someone else	0.73	0.03
		(11) Having strong negative feelings, e.g., fear, horror, anger,	0.85	0.02
		(12) Loss of interest in activities that you used to enjoy	0.82	0.02
		(13) Feeling distant or cut off from other people	0.82	0.02
		(14) Trouble experiencing positive feelings	0.81	0.02
		(15) Irritable behavior, angry outbursts, or acting aggressively	0.73	0.03
		(16) Taking too many risks	0.54	0.04
		(17) Being “superalert” or watchful or on guard	0.64	0.03
		(18) Feeling jumpy or easily startled	0.75	0.03
		(19) Having difficulty concentrating	0.77	0.02
		(20) Trouble falling or staying asleep	0.67	0.03
Shame	SSGS	(1) I want to sink into the floor and disappear	0.79	0.03
		(3) I feel small	0.77	0.03
		(5) I feel that I am a bad person	0.83	0.02
		(7) I feel humiliated, disgraced	0.80	0.02
		(9) I feel worthless, powerless	0.82	0.02
Guilt	SSGS	(2) I feel remorse, regret	0.76	0.03
		(4) I feel tension about something I have done	0.85	0.02
		(6) I cannot stop thinking about something bad I have done	0.90	0.01
		(8) I feel like apologizing, confessing	0.75	0.03
		(10) I feel bad about something I have done	0.88	0.02
Factor Correlations		Betrayal with perpetration	0.63	0.06
		Shame with guilt	0.82	0.05
Modifications		*Correlated Items*		
		PCL-5 item 18 with PCL-5 item 17 PCL-5 item 7 with PCL-5 item 6 PCL-5 item 2 with PCL-5 item 3 PCL-5 item 5 with PCL-5 item 4 PHQ-9 item 4 with PHQ-9 item 3 PCL-5 item 20 with PHQ-9 item 3 MIES item 2 with MIES item 1 MIES item 4 with MIES item 3 MIES item 6 with MIES item 5 MIES item 8 with MIES item 7	0.64 0.62 0.54 0.56 0.40 0.49 0.60 0.65 0.76 0.48	0.03 0.04 0.04 0.04 0.05 0.04 0.04 0.05 0.04 0.07

*All reported loadings are standardized. MIES, Moral Injury Event Questionnaire; SSGS, State Shame and Guilt Survey; PCL-5, Posttraumatic Stress Disorder Checklist-5; PHQ-9, Patient Health Questionnaire-9. All reported loadings are significant at p < 0.001*.

### Structural model testing combat and MST within moral injury framework

Overall, the structural model provided good fit to the data, $X_{\left(1147\right)}^{2}$ = 3040.18, *p* < 0.001. RMSEA = 0.07, CFI = 0.86, TLI = 0.85, SRMR = 0.07 (see Figure 1). We found mixed support for our primary pathways. MST was indirectly associated with PTSD-depression via betrayal (β = 0.10, *p* < 0.01, 95% CI = 0.04 −0.20) and combat was indirectly associated with PTSD-depression via perpetration (β = 0.07, *p* < 0.05, 95% CI = 0.02 −0.13). The indirect path from combat through shame to PTSD/depression was significant (β = 0.16, *p* < 0.01, 95% CI = 0.07 −0.27) although the path through guilt was not (β = −0.04, *p* = 0.20, 95% CI = −0.13 −0.02). Neither of the indirect paths from MST to PTSD/depression via moral injury mechanisms were significant (via shame, β = 0.10, *p* = 0.08, 95% CI = −0.003 −0.23; via guilt, β = −0.003, *p* = 0.81, 95% CI = −0.05 −0.01). The specific indirect paths from combat to perpetration to shame to PTSD-depression was not significant (β = 0.06, *p* = 0.06, 95% CI = 0.02 −0.16) and neither was the path through perpetration to guilt to PTSD/depression (β = −0.01, *p* = 0.23, 95% CI = −0.01 −0.003). Contrary to expectation, combat was not indirectly associated with PTSD-depression via betrayal (β = 0.04, *p* = 0.07, 95% CI = 0.01 −0.11). See Figure 1 for all significant and non-significant direct paths.

## Discussion

This study's purpose was to test a frequently cited model of moral injury (1): whether MST and combat were associated with PTSD and depression via perpetration-based morally injurious events or betrayal-based morally injurious events and subsequent guilt and/or shame. Our critical test accounted for pathways suggested by the broader trauma literature; namely, that guilt and shame may contribute to PTSD and depression independent of perpetration or betrayal. We found mixed support for the key pathways of the moral injury model. Betrayal was a significant pathway from MST to PTSD-depression and perpetration was a significant pathway from combat to PTSD-depression; unexpectedly, betrayal did not have an indirect effect from combat to PTSD-depression. Shame, but not guilt, accounted for some of the association between combat and PTSD-depression.

### Betrayal

Until this study, MST's betrayal aspects have not been studied using the moral injury framework, but have been conceptualized within the institutional betrayal literature. Institutional betrayal refers to “when institutional action or inaction exacerbates the impact of traumatic experiences (p. 577)…or causes harm to an individual who trusts or depends upon that institution” [p. 578 (41)]. Thus, the definition of institutional betrayal is reminiscent of the moral injury field's definition of betrayal, i.e., “betrayal of what's right in a high stakes situation *by a trusted authority figure*” (2, 42). Consequently, MST may be a prototypical example of several key facets of both institutional betrayal and moral injury betrayal: failure to protect service members dependent on the military, disruption of belongingness in a close community by interpersonal violence, and institutional priorities that run counter to prosecuting sex crimes (43). In a sample of 49 male and female veterans, the majority perceived their MST as involving institutional betrayal and, notably, perceptions of institutional betrayal significantly predicted PTSD and depressive symptoms (44). To our knowledge, Monteith et al. is the only study that examined MST within the institutional betrayal framework. This suggests a largely unexplored and potentially fruitful lens through which to deepen understanding of and treatment for MST and the betrayal-like aspects of moral injury.

Combat was directly associated with betrayal, and betrayal was associated with PTSD-depression; however, combat was not associated with PTSD-depression via betrayal. The betrayal aspects of combat were originally articulated as a reaction to situations in which service members found themselves perpetrating morally injurious acts, for instance, being sent to fight a war they perceived as unjust or unlawful, being sent to fight with inadequate weaponry (i.e., being sent to die), or being ordered to carry out unlawful actions (42). It may be that some combat traumas have both a betrayal-based morally injurious component—being disturbed by the consequences of leadership decision-making—as well as an institutional betrayal component—feeling that one's trust and dependency on the military was violated. Future research should test alternative conceptualizations of betrayal within the moral injury framework, such as testing betrayal as a moral injury outcome, or testing the institutional betrayal model more directly within the moral injury framework.

Betrayal was not directly associated with either guilt or shame. We speculate that betrayal may evoke reactions such as anger or self-disgust that were not directly assessed in the current study and that have known relations with PTSD and depression. In support of this hypothesis, a previous study of combat-deployed Marines found a significant direct association between betrayal and anger and an indirect association with PTSD via anger (25). Similarly, in a recent study of Israeli combat veterans, betrayal-based morally injurious events were associated with symptoms of posttraumatic stress, depressive attributions, and self-disgust (45). Thus, these results suggest that additional “mechanisms” of moral injury such as anger, rage, and disgust should be examined in future studies and potentially targeted in treatment.

### Perpetration

Perpetration accounted for the association between combat and PTSD-depression and was associated with both shame and guilt, although the specific indirect paths from combat to perpetration to shame or guilt to PTSD-depression were not significant. Three previous studies have found a path from prototypical perpetration-type combat events (e.g., killing, atrocities) to negative mental health outcomes through combat-related guilt (23, 24, 26); however, these studies did not assess shame alongside guilt. Similar to our finding of a significant association between shame and PTSD-depression but not guilt and PTSD-depression, one of the few studies that examined both guilt and shame in combat veterans found that shame-proneness was positively associated with PTSD, but guilt-proneness was negatively associated with PTSD (46). In general, previous studies have examined just combat-related guilt or guilt-proneness. Our study is one of the first to compare the relative contribution of guilt vs. shame, and also to account for individual differences in veterans' appraisals of combat as perpetration-type morally injurious events.

We also found evidence for a direct path from combat to guilt distinct from appraisals of betrayal or perpetration. Clinical literature has described the ways that combat-related guilt can function: as an “honoring” impulse so that people who were killed or wronged are not forgotten, or, as a way of assuming responsibility and thus lessening one's sense of helplessness after uncontrollable or chaotic situations (47, 48). A sufficient conceptual and empirical model of moral injury must account for the non-specific occurrence of guilt and shame following traditional life-threat traumas as well as morally injurious events. Future models of moral injury also need to account for guilt and shame's cumulative effects as well as their unique effects.

The role of MST within the moral injury framework needs additional theoretical consideration and clarification. The current study established that experiencing MST may be a betrayal-based morally injurious event that may benefit from a moral injury-focused intervention approach. At the same time, *perpetrating* MST, such as rape of civilians or fellow service members, falls well within the domain of potential perpetration-based morally injurious event that could lead to moral injury. Including both experiencing and perpetrating MST within the morally injurious events domain could raise complicated theoretical issues and troubling clinical scenarios. In terms of theory, currently, the moral injury model does not consider guilt and shame as necessarily irrational or dysfunctional responses to morally injurious events. A consequence of including experiencing MST within the morally injurious events category will be to make the moral injury model agnostic as to whether guilt and shame are appropriate or inappropriate responses to morally injurious events. In terms of clinical approaches, at face value, guilt, shame, self-disgust, and rage in response to perpetrating MST would necessitate a different treatment approach than guilt, shame, self-disgust, and rage in response to the betrayal of experiencing MST. Moreover, there would be potential for iatrogenic harm if MST survivors were treated in the same clinical settings as MST perpetrators. Thus, how MST fits into the moral injury domain remains both a pressing theoretical and clinical concern for the field.

Strengths of the study included the two-time point design, which diminished potential for ambient measurement variance contributing to significant results, and psychometrically strong measures of hypothesized constructs. However, interpretation of results should be tempered by study limitations. Secondary data analysis limited the range of tested moral injury mechanisms. For example, the parent study did not include measures of anger or disgust, which may be additional intervening variables between betrayal and PTSD-depression (25, 45). We had relatively lower endorsement of MST compared to combat, and thus future studies may benefit from oversampling for veterans who have experienced MST. The parent study did not directly assess perceptions of institutional betrayal. Future studies should include a measure of institutional betrayal, which could clarify the relation between perpetration and betrayal and moral injury outcomes; this is relevant given that our sample was comprised of veterans who were willing to be enrolled in the local VA healthcare system, which may limit generalizability. Specifically, some veterans may not be willing to seek VA services due to feelings of institutional betrayal, and thus, future research should include a broader sample of veterans who seek services within and outside of VA. The combat exposure variable oversampled potentially fear-based experiences (e.g., experiencing incoming rocket attacks) and under sampled for potentially morally injurious acts (e.g., failing to prevent atrocities) and so direct and indirect paths from combat exposure to moral injury-related variables may have been attenuated. Lastly, this study did not include a pre-trauma assessment and thus was not designed to test prospective predictive relations among study variables.

This study contributes to boundary clarification of the moral injury construct, suggests potentially modifiable mechanisms of moral injury that can become treatment targets or guide the development of moral injury-focused psychotherapies, and points to the institutional betrayal literature as a novel and complementary framework for studying and treating moral injury. The moral injury field is evolving and going through the normative process of boundary setting and construct formation. This current movement is similar to the movement in the PTSD/trauma field during the DSM-IV-TR revision to the PTSD diagnosis when the field debated what constitutes a traumatic event, how to conciliate objective and subjective definitions of trauma, and whether “bracket creep” (i.e., expanding the definition of trauma) is a problem and how to handle it [e.g., (49)]. Our hope is that this current study poses directions for future research that can continue to assist clinicians and researchers in identifying and testing potential mechanisms of moral injury development and treatment.

## Author contributions

SF conducted the analyses and wrote the first draft of the manuscript. EM, SM, BD, and NK designed the study and acquired funding. All authors provided feedback (SF, BD, SM, NK, HLB, EM) provided feedback on early idea development, contributed to revising the manuscript, and have approved the final article.

### Conflict of interest statement

The authors declare that the research was conducted in the absence of any commercial or financial relationships that could be construed as a potential conflict of interest. The handling editor declared a shared affiliation, though no other collaboration, with one of the authors NK.
